# Toe Gap Force is Related to Ultrasonic Parameter of Calcaneus Bone Status in General Population

**DOI:** 10.2174/1874325001812010024

**Published:** 2018-01-30

**Authors:** Tsuyoshi Tajika, Atsushi Yamamoto, Noboru Oya, Takanori Kitagawa, Hiroki Kobayashi, Hitoshi Shitara, Haku Iizuka, Kenji Takagishi, Hirotaka Chikuda

**Affiliations:** 1 Department of Orthopaedic Surgery, Gunma University Graduate School of Medicine, 3-39-22, Showa-machi, Maebashi, Gunma, 371-8511, Japan; 2 Keiyu Orthopaedic Hospital,1741 Hanetsuku Tatebayashi, Gunma, 374-0011, Japan; 3 Department of Orthopaedic Surgery, Saint-Pierre Hospital 786-7, Kamisano-machi, Takasaki, Gunma, 370-0857, Japan

**Keywords:** Bone health, Calcaneal speed of sound, Hand grip strength, Toe-gap force strength, Hip abductor muscle strength, Key pinch strength

## Abstract

**Background:**

Elucidating the relation between bone health condition and muscle strength can provide useful knowledge for Japan’s super-aged society. This study ascertained the Speed Of Sound (SOS) of the calcaneus and upper and lower limb muscle strength in a general population.

**Methods:**

Medical examinations were conducted of 277 adult residents (100 men, 177 women; average age 67.1 years) of a mountain village. Bilateral hand grip and hand key pinch strength were measured. Hip abductor muscle strength was measured using a hand-held dynamometer. The pressure between the hallux and second toe (toe-gap force) was measured using a toe pressure measuring instrument. The Speed Of Sound (SOS) of the calcaneus was assessed using an ultrasound bone densitometer. Stepwise linear regression was used to identify the predictor of SOS using several factors as explanatory variables: gender, age, height, weight BMI, grip and key pinch power strength, hip abductor muscle strength, and toe-gap force in bilateral side. P values of < .05 were inferred as statistically significant.

**Results:**

Significant positive correlation was found between the SOS and each of bilateral hand grip and hand key pinch strength, hip abductor muscle strength, and toe-gap force in all participants. Stepwise logistic regression revealed gender (β coefficient= -0.32, p= .0001), age (β coefficient=-0.53, p= .000), height (β coefficient= -0.19, p= .03), and right toe-gap force (β coefficient= 0.11, p= .027) as predictors of calcaneal SOS for all participants.

**Conclusion:**

Toe-gap force assessment might be more useful to predict calcaneal SOS than grip assessment in the general population.

## 1 INTRODUCTION

With the rapidly changing demographic structure of Japan, a country with increasing trends toward general population aging, osteoporosis poses a major public health problem. The estimated number of people in Japan with osteoporosis was 12.8 million in 2012 [1]. Low Bone Mineral Density (BMD) with increased fall incidence caused higher fracture risk [2]. To prevent the progress of osteoporosis and osteoporosis-related fractures, it is important to check bone status regularly and to assess bone-health-related factors among the general population. Some earlier reports have described a significant correlation between BMD and muscle strength [3-7]. Hand grip strength is positively correlated with the BMD of the proximal femur or BMD of lumbar vertebrae [3, 4]. Several reports have described a positive correlation between hand grip strength and the BMD of the radius [5-7]. These results suggest that the relation between muscle strength and BMD was not merely site-specific, but systemic. Quantitative ultrasound (QUS) assessment of the calcaneus rapidly provides a risk assessment of osteoporosis [8]. QUS was used to screen the bone quality in the general population in many studies [9-11]. An earlier report described that plantar flexion force is related significantly to calcaneus SOS in postmenopausal women [12]. However, few reports describe investigations of the relation between the calcaneus SOS and upper and lower extremity muscle strength. It seems to be useful to recognize the relevant physical factors as a determinant of bone status for the progress of preventative strategy of osteoporosis. This study used QUS to assess the association between SOS of the calcaneus and upper and lower extremity muscle strength. Moreover, this study investigated the predictive role of muscle strength on SOS of the calcaneus in mountain village residents.

## 2 MATERIALS AND METHODS

Local medical examinations intended for early detection of cancer and for prevention of lifestyle-related diseases are administered regularly for residents of a mountain village in Japan, where agroforestry and tourism are the main industries. For this study, 277 people (100 men, 177 women; average 67.1 years of age, range 30-89) were selected randomly as candidates. The exclusion criteria applied to candidates were the following: (1) any prior operation in bilateral hand or hip or foot; (2) any pain of bilateral upper and lower extremity; and (3) any use of medication affecting bone metabolism, such as bisphosphonates and glucocorticoids. (4) the subjects who with conditions that possibly cause secondary osteoporosis such as endocrine, neoplastic, genetic and collagen disease. After they had been informed of the study protocol and had been told that their data would be published, all candidates gave their consent to participate in this study. This study was approved by the regional ethics board.

### 2.1 Anthropometric Measurements

A&D Corp., Tokyo, Japan). Weight was measured using a multi-frequency segmental body composition analyzer (MC780U; Tanita Corp., Tokyo, Japan).

### 2.2 Grip and Key Pinch Strength Measurements

A digital dynamometer (Takei Scientific Instruments Co. Ltd., Tokyo, Japan) was used to measure grip strength. A pinch gauge (MG-4320NC pinch gauge; B & L Engineering, Santa Ana, California, United States) was used to measure the key pinch of both sides. Grip testing was conducted using the standard position recommended by the American Society of Hand Therapists. Key pinch testing was performed with the shoulder, elbow, forearm, and wrist in a neutral position. Key pinch is the thumb pad to the lateral aspect of middle phalanx of index finger. For each grip and pinch test, three measurements were performed on bilateral sides. All tests were administered by a single orthopedic surgeon.

### 2.3 Hip Abductor Isometric Strength Measurements

**Fig. (1):**
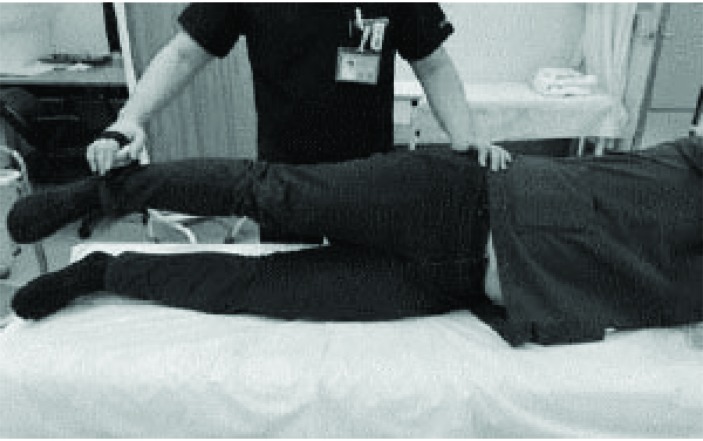
Measurements of isometric strength of hip abductor on leg.

Measurements of isometric strength of hip abductor on both legs were made using a handheld dynamometer (MICRO FET2TM; Hoggan Health Industries, Salt Lake City, UT, USA). Hip abduction strength was measured with subjects positioned side-lying on a mat with the hip and knee in a neutral position. The tester applied the handheld dynamometer 5 cm proximal to the lateral malleolus to oppose the participant’s efforts to sustain the position of a raised limb [13]. Measurement of hip abductor strength was performed once on the bilateral side (Fig. **1**).

### 2.4 Toe-Gap Force Measurements

**Fig. (2):**
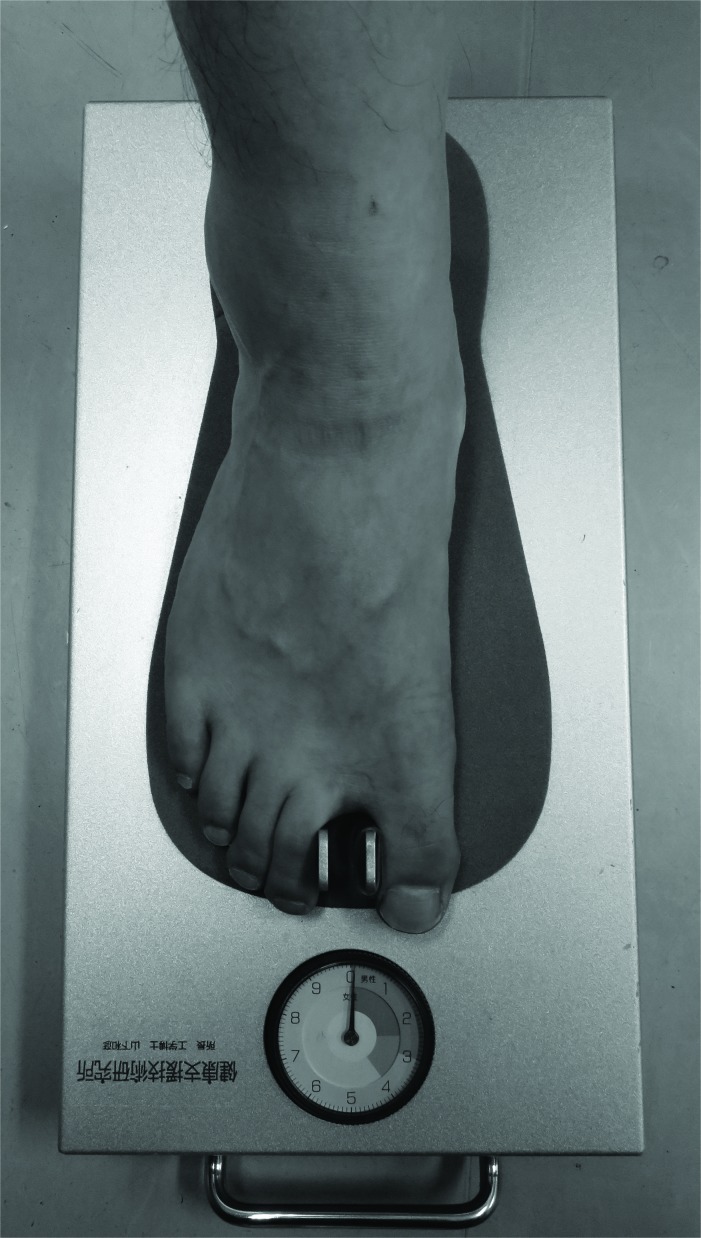
Measurements of toe-gap force gauge by using Checkerkun ^TM^.

We used toe-gap force gauge (Checkerkun NI Industrial Co. Ltd, Ageo, Japan) (Fig. **2**). A foot stand is provided with a fixed measuring part, a movable measuring part, and a display part. By strongly grasping the fixed measuring part and the movable measuring part between the first toe and second toe, the pressure between them can be measured and displayed [14]. Measurements of toe-gap force were repeated twice for the right and left foot. Either bigger value of two measurements was adopted in each side.

### 2.5 Quantitative Ultrasound Assessment of Calcaneus

**Fig. (3):**
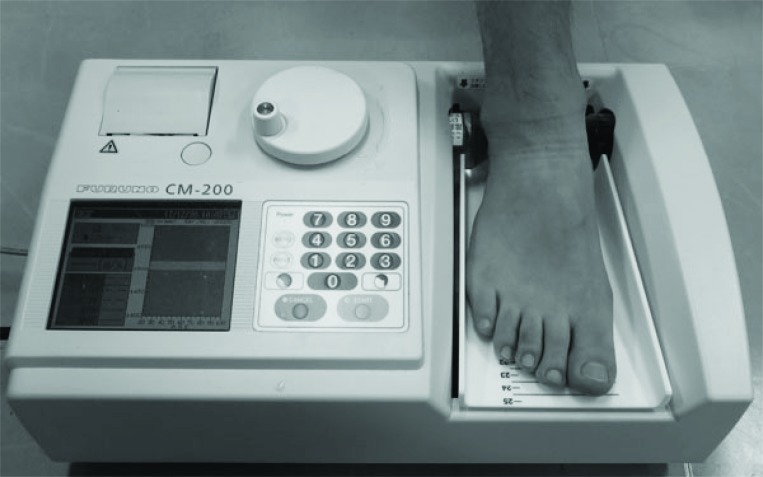
Measurements of quantitative ultrasound assessment of calcaneus by using CM-200 ^TM^.

The SOS of the calcaneus bone of right side was measured using an ultrasound bone densitometer with a gel coupled system (CM-200; Furuno Electric Co. Ltd., Nishinomiya, Japan) (Fig. **3**). A height adjustable footplate of this device accurately can be aligned a different size heel to the optimized position.The sound wave passes from one transducer located in the fixed cylinder through the participant’s heel to another transducer in the movable cylinder. The unique sensor of this device can compensate for the participant’s heel temperature and can thereby provide accurate SOS measurements. This machine was calibrated with a physical phantom. Precision error (percent coefficient of variation) using the phantom technique was 0.15% and was 0.27% [15]. All measurements were taken by the same orthopedic surgeon.

### 2.6 Statistical Analyses

Differences in the values of the indices were assessed for significance using analysis of variance for comparisons among multiple groups. A Steel-Dwass test was used for pairs of respective age groups. Spearman's rank correlation coefficient was calculated to elucidate the relation between SOS and age, height, weight, Body Mass Index (BMI), bilateral grip and key pinch strength, hip abductor muscle strength, and toe grip strength. Stepwise logistic regression models, with exclusion of covariates with univariate p values greater than 0.2 was used to identify the predictors of SOS using gender, age, height, weight, BMI, gender, bilateral grip and key pinch strength and hip abductor muscle strength, toe grip strength as explanatory variables. A P value of < .05 was regarded as statistically significant.

## 3 RESULTS

Table **1** presents characteristics of the participants including anthropometry variables, strength of the respective muscles, and SOS. The SOS of men showed significant positive correlation with each of weight and bilateral grip and key pinch strength, hip abductor muscle strength, and toe-gap force. Positive correlation was found for the SOS of women with each of weight, height, bilateral grip and left key pinch strength, bilateral hip abductor muscle strength, and left toe-gap force. For all participants, significant positive correlation was found between the SOS and each of bilateral grip and key pinch strength, hip abductor strength, and toe-gap force (Table **2**).

In men, stepwise linear regression analysis revealed that age and right hip abductor muscle strength and right toe-gap force were significant contributors to calcaneal SOS. The coefficient of determination (R2) for this stepwise linear regression model was 0.37. For women, stepwise linear regression analysis revealed age as a significant contributor to calcaneal SOS. For this stepwise linear regression model, R2 was 0.34. In all participants, gender and age and height and right toe-gap force were predictors of calcaneal SOS. For this stepwise linear regression model, R2 was 0.39 (Table **3**).

## 4 DISCUSSION

This study assessed the association between calcaneal SOS and upper and lower muscle strength. Stepwise linear regression revealed that gender, age, height, and right toe-gap force were predictors of calcaneal SOS in all participants. Yoshimura *et al*. demonstrated that multivariate regression analysis showed a significant association between osteoporosis and sarcopenia occurrence within four years (odds ratio, 2.99; 95% confidence interval, 1.46–6.12; p < .01) in a four-year follow-up study that examined 1099 subjects aged ≥60 years [16]. It is extremely important to recognize the close relation between osteoporosis and muscle strength in a super-aged society such as that of Japan. Our study identified, gender difference was as a predictor of calcaneal SOS. Prior earlier studies have demonstrated that gender difference might alter the relation with muscle strength and BMD [17, 18]. Nguyen *et al*. found that reported that quadriceps strength predicted bone density at the proximal femur in elderly men but not in women in 709 elderly men and 1080 women [18]. Bevier *et al*. demonstrated muscle strengths was as a more important predictor of BMD more in men than in women [19]. In our study, stepwise linear regression analysis found right hip abductor muscle strength and right toe-gap force were significant predictors to calcaneal SOS in men, however, no muscle strength in each part contributed to calcaneal SOS in women as the result of previous studies (17.18). Prior report suggested that differences in hormonal status, body composition, degree of physical activity, and muscle contractile characteristics, may might cause explain the gender differences in relation with muscle strength and bone status [19]. Some studies reported a correlation between muscle strength and circulating estrogen levels [20]. Estrogen receptors are present in muscle membrane and a greater number of estrogen receptors in muscle fibers was demonstrated in adult men and women as compared to postmenopausal women [21]. These receptors might contribute to the synthesis of muscle tissue at rest and the repairing process of muscle fibers after exercise [22]. We did not measure the serum estrogen level in this study. Estrogen level might cause the gender difference in the relation with bone status and muscle strength. Further additional studies should be conducted be assessed the underlying factors for of these gender differences.

We investigated the grip strength and key pinch strength as indicators of upper limb muscle strength and measured the hip abductor muscle strength and toe-gap force as indicators of lower limb muscle strength. Handgrip strength assessment has been used as a reliable index for whole body muscle strength [23]. Earlier studies have evaluated the association between grip strength and BMD of the spine and femur and radius [3-7]. Key pinch strength reflecting the intrinsic muscles of the hand is measured easily, just as it is for the handgrip. Our earlier cross-sectional report described non-dominant key pinch strength as a significant predictor of calcaneal SOS in the general population [24]. Muscle strength of hip abductor was assessed to clarify the relation with BMD of the femur [25]. We investigated the toe-gap force measurement as a new index of lower limb muscle strength. Yamashita *et al*. developed the toe-gap force measurement device to evaluate lower extremity muscle strength, which has a role in efforts to prevent falls by elderly people [14]. The clipping toe-gap force is measured between the great-toe and the second toe. The toe-gap force in this situation is generated by the collaboration of the flexor-tensor muscles of the lower limbs. They reported that assessment of toe-gap force in lower limbs is comparably important to assessment of handgrip in upper limbs. Few reports have described studies of the relation with upper and lower muscle strength and calcaneal SOS. Therefore, several aspects remain unclear. Prior earlier studies have demonstrated that skeletal loading such as strain and muscular traction forcing influences bone density and bone quality. Aydin *et al*. reported a significant positive correlation between the BMD of the forearm and hand grip strength at bilateral handgrip strength in 234 men with various rheumatic complaints [7]. Moreover, Mayoux-Benhamou *et al*. used quantitative ultrasound to examine 45 healthy postmenopausal women and thereby evaluate the relation between plantar flexion strength produced by contraction of gastrocnemii-soleus muscle and calcaneal SOS [12]. They demonstrated that plantar flexion strength is associated with calcaneal SOS (r = 0.3, P = .04) and that it is a predictor of calcaneal SOS (R2= 9%). Bayramoğlu *et al*. demonstrated from a study of 62 postmenopausal women that the isokinetic strength of hip abductors is significantly correlated with femoral BMD [26]. The voluntary loading of muscles attached to the bone might affect the mechanical stress on bone density site-specifically.

Several reports have described an association between handgrip strength and BMD and bone status other than forearm bones [3, 27]. Sinaki *et al*. demonstrated a significant positive relation between hand grip strength and BMD of lumbar vertebrae and proximal femur in healthy premenopausal Caucasian women [3]. Marin *et al*., reported that handgrip strength was most importantly associated to the BMD of the lumbar spine, femoral neck, and total body (lumbar spine, r=0.49, p< .001; femoral neck, r=0.56, p< .001; and total body, r=0.52, p< .001) in 117 physically active postmenopausal women [27]. These results suggest that the effect of handgrip strength on BMD is systemic. In our study, calcaneal SOS was correlated positively with bilateral handgrip and key pinch strength, hip abductor strength, and toe-gap force. Of all muscle strength tests, stepwise linear regression analysis found that right toe-gap force was a significant predictor of calcaneal SOS for all participants. The osteogenic response appears to saturate after external loading. Loading on the calcaneal bone might produce the intrinsic muscles attached around the calcaneus. It might be more effective to check toe-gap force when screening the degree of calcaneal SOS.

Our study had several limitations. First, this study is cross-sectional. Rhodes *et al*., in a prospective study of 44 elderly women, demonstrated the positive effects of one year of progressive resistance exercise on dynamic muscular strength and its relation to lumbar and femur BMD [28]. Prospective investigations must be undertaken to elucidate the relation between toe-gap force and calcaneal SOS. Second, we were unable to assess the association between weight-bearing exercise and calcaneal SOS. The role of weight bearing against gravity was emphasized by Vico *et al*., who investigated the effects of mechanical stimulus to bone remodeling on seven foot bones of male rats exposed to 7 days of weightlessness during space flight [29]. Moreover, reportedly, physical activity is associated with calcaneal SOS. Weight-bearing exercise more strongly affects the maximization of peak bone mass than non-weight bearing exercise does [30]. Third, several important factors may affect muscle strength including gonadal status, nutritional status [22]. We could not assess these items. Additional studies must be conducted to elucidate the quantitative ultrasound assessment with multiple related factors.

In conclusion, stepwise linear regression analysis revealed that age and right hip abductor muscle strength and right toe-gap force were significant contributors to calcaneal SOS in men. In women, stepwise linear regression analysis revealed age as a significant contributor to SOS. In all participants, gender, age, height and right toe-gap force strength were found to be predictors of calcaneal SOS. Toe-gap force assessment might be more useful as a tool for bone health assessment.

## Figures and Tables

**Table 1:** Characteristics of participants according to age cluster.

Age group	Total examined member	Height (cm)	Weight (kg)	Body Mass Index (g/cm^2^)	Right Grip (kg)	Center grip (kg)	Right key pinch (kg)	Center key pinch (kg)	Right hip abductor (kg)	Center hip abductor (kg)	Right toe-gap force (kg)	Center toe-gap force (kg)	Calcaneus of speed sound
Men													
Age													
<50	7	168.7 (5.1) ^d,e^	64.0 (10.3)	22.4 (3.2)	42.5 (8.0) ^d,e^	42.2 (6.9) ^d,e^	10.7 (1.9) ^e^	9.9 (2.0) ^e^	38.7 (12.7) ^e^	37.3 (11.9) ^e^	3.6 (0.9)	3.3 (0.8)	1547.3 (35.1) ^c,d,e^
50–59	11	166.7 (6.6) ^e^	69.2 (7.5) ^d,e^	24.8 (1.5)	36.5 (9.5) ^e^	38.6 (8.3) ^e^	9.9 (2.7)	10.3 (1.7)^e^	31.8 (7.2) ^e^	32.1 (3.8) ^e^	3.4 (1.4)	2.8 (1.3)	1513.9 (29.1) ^e^
60-69	34	164.2 (5.2) ^e^	64.9 (9.7)	24.0 (3.2)	36.8 (6.4) ^d^	36.4 (7.6) ^e^	9.7 (1.9) ^e^	9.3 (1.8) ^e^	30.0 (7.8) ^e^	31.5 (7.3) ^e^	3.1 (1.1)	2.9 (0.9) ^e^	1504.6 (26.5) ^e^
70-79	26	160.3 (5.9)	61.2 (8.3)	23.8 (2.7)	32.4 (7.3) ^e^	32.0 (7.2) ^e^	9.1 (1.7)	8.6 (1.9)	27.4 (7.2)	27.9 (8.1)	2.9 (0.9)	2.6 (1.0)	1503.8 (26.7) ^e^
80≤	22	159.1(4.4)	58.9 (8.6)	23.2 (2.8)	26.5 (4.3)	24.6 (6.0)	7.8 (1.7)	7.4 (1.4)	23.8 (6.1)	24.5 (5.3)	2.5 (1.4)	2.0 (1.1)	1480.0 (18.7)
Total	100	162.6 (6.2)	63.0 (9.3)	23.8 (2.9)	33.7 (8.1)	33.3 (8.9)	9.2 (2.1)	8.7 (2.0)	31.1 (24.5)	31.9 (24.6)	3.0 (1.2)	2.6 (1.1)	1503.0 (30.3)
Women	–	–	–	–	–	–	–	–	–	–	–	–	–
<50	16	154.4 (4.4) ^d,e^	52.1 (8.4)	21.8 (3.0)	24.9 (5.9) ^d,e^	25.1 (6.0) ^d,e^	6.9 (1.1) ^e^	6.9 (1.1) ^d,e^	22.1 (7.6)	22.5 (5.3) ^d^	2.3 (0.9)	2.2 (0.8)	1530.3 (41.4) ^b,c,d,e^
50-59	29	156.6 (6.8) ^d,e^	53.7 (7.9) ^d^	21.9 (2.8)	23.8 (3.2) ^d,e^	22.6 (3.2) ^d,e^	6.6 (1.2) ^e^	6.4 (1.0) ^e^	21.1 (4.1)	22.1(5.7) ^d^	2.4 (1.0)	2.1 (0.9)	1496.2 (34.3) ^d,e^
60-69	56	152.7 (5.3) ^d,e^	55.3 (9.6) ^d^	23.7 (3.4)	21.4 (5.2) ^e^	20.9 (5.1) ^e^	6.3 (1.4)	6.1 (1.3)	20.5 (5.4)	21.7 (6.5)	2.5 (1.1)	2.1 (1.1)	1482.5 (20.7) ^e^
70-79	56	147.6 (4.9)	48.9 (6.2)	22.4 (2.5)	19.7 (3.7) ^e^	19.0 (3.2) ^e^	6.1 (0.9)	5.8 (1.1)	19.1 (4.8)	18.4 (4.3)	2.3 (1.2)	2.1 (1.2)	1474.6 (22.1)
80≤	20	145.1 (6.5)	49.7 (7.7)	23.6 (2.9)	16.7 (2.9)	15.8 (2.7)	5.4 (1.3)	5.2 (1.2)	18.7 (5.3)	18.5 (5.9)	2.0 (1.0)	1.7 (0.8)	1460.7 (13.5)
Total	177	151.0 (6.7)	52.1 (8.4)	22.8 (3.0)	21.0 (4.9)	20.4 (4.8)	6.2 (1.3)	6.0 (1.2)	20.1 (5.3)	20.4 (5.8)	2.3 (1.1)	2.1 (1.0)	1484.1 (30.8)

Mean values are shown with the standard deviation.

SD: Standard Deviation

a. Significantly different (p< .05) from values of the younger-than-50-years-old age group

b. Significantly different (p< .05) from values of the 50s age group

c. Significantly different (p< .05) from values of the 60s age group

d. Significantly different (p< .05) from values of the 70s age group

e. Significantly different (p< .05) from values of the older than 80 years old age group

**Table 2:** Correlation between calcaneus SOS and anthropometric and upper and lower extremity muscle strength.

Variable	Men	*p*	women	*p*	All participants	*p*
Age	-0.45	0.000	-0.53	0.000	-0.43	0.000
Height	0.17	0.090	0.28	0.000	0.38	0.000
Weight	0.22	0.030	0.22	0.003	0.36	0.000
Body mass index	0.18	0.090	0.09	0.244	0.16	0.007
Right grip	0.42	0.000	0.32	0.000	0.46	0.000
Center grip	0.40	0.000	0.3	0.000	0.45	0.000
Right key pinch	0.31	0.003	0.15	0.052	0.36	0.000
Center key pinch	0.39	0.000	0.2	0.006	0.40	0.000
Right hip abductor	0.37	0.000	0.16	0.034	0.37	0.000
Center hip abductor	0.41	0.000	0.2	0.009	0.40	0.000
Right toe -gap force	0.34	0.001	0.17	0.024	0.31	0.000
Center toe-gap force	0.30	0.003	0.14	0.062	0.27	0.000

**Table 3:** Predictors of calcaneal SOS.

Predictors	β coefficient (SE)	*p*	*R* ^2^
In men	–	–	–
Age	-0.37 (0.24)	0.0001	0.37
Right toe-gap force	0.19 (2.2)	0.024	
center hip abductor	0.22 (0.37)	0.024	–
In women	–	–	–
Age	-0.61 (0.19)	0.00	0.34
In all participants	–	–	–
Gender	-0.31 (5.1)	0.0001	0.40
Age	-0.53 (0.16)	0.00	–
Height	-0.18 (0.32)	0.028	–
Right toe-gap force	0.11(1.4)	0.027	–

## LIST OF ABBREVIATIONS

BMD: Bone Mineral Density
BMI: Body Mass Index
QUS: Quantitative Ultrasound
SOS: Speed Of Sound
